# A bivalent compound targeting CCR5 and the mu opioid receptor treats inflammatory arthritis pain in mice without inducing pharmacologic tolerance

**DOI:** 10.1186/s13075-018-1661-5

**Published:** 2018-07-27

**Authors:** Raini Dutta, Mary M. Lunzer, Jennifer L. Auger, Eyup Akgün, Philip S. Portoghese, Bryce A. Binstadt

**Affiliations:** 1 Department of Pediatrics and Center for Immunology, University of Minnesota, 2-114 Wallin Medical Biosciences Building, 2101 6th Street SE, Minneapolis, MN 55414 USA; 2 Department of Medicinal Chemistry, College of Pharmacy, University of Minnesota, Minneapolis, MN USA

**Keywords:** Analgesia, CCR5, Chemokine receptor, Heteromer, Inflammation, Opioid receptor, Pain, Rheumatoid arthritis

## Abstract

**Background:**

Pain accompanies rheumatoid arthritis and other chronic inflammatory conditions and is difficult to manage. Although opioids provide potent analgesia, chronic opioid use can cause tolerance and addiction. Recent studies have demonstrated functional interactions between chemokine and opioid receptor signaling pathways. Reported heterodimerization of chemokine and opioid receptors led our group to develop bivalent compounds that bind both types of receptors, with the goal of targeting opioids to sites of inflammation. MCC22 is a novel bivalent compound containing a CCR5 antagonist and mu opioid receptor (MOR) agonist pharmacophores linked through a 22-atom spacer. We evaluated the efficacy of MCC22 in the K/B.g7 T-cell receptor transgenic mouse model of spontaneous inflammatory arthritis.

**Methods:**

MCC22 or morphine was administered intraperitoneally at varying doses to arthritic K/B.g7 mice or nonarthritic control mice. Mechanical pain hypersensitivity was measured each day before and after drug administration, using the electronic von Frey test. The potency of MCC22 relative to that of morphine was calculated. Functional readouts of pain included grip strength and nesting behavior. A separate dosing regimen was used to determine whether the drugs induced pharmacologic tolerance.

**Results:**

MCC22 provided ~ 3000-fold more potent analgesia than morphine in this model. Daily treatment with MCC22 also led to a cumulative analgesic effect, reducing the daily baseline pain level. MCC22 produced no observable analgesic effect in nonarthritic control mice. Importantly, repeated administration of MCC22 did not induce pharmacologic tolerance, whereas a similar regimen of morphine did. Both grip strength and nesting behaviors improved among arthritic mice treated with MCC22. Ankle thickness and arthritis scores were not affected by MCC22. The analgesic effect of MCC22 was abolished in K/B.g7 mice genetically lacking CCR5, demonstrating the receptor specificity of the antagonist pharmacophore.

**Conclusions:**

MCC22 is a novel bivalent ligand that targets CCR5 and MOR. Our findings demonstrate that MCC22 provides highly potent analgesia and improved functional outcomes in a model of inflammatory arthritis, without inducing typical opioid tolerance. These findings suggest that MCC22 or similar compounds could be used to treat the pain associated with inflammatory arthritis and related conditions, while minimizing the risks typically associated with chronic opioid use.

**Electronic supplementary material:**

The online version of this article (10.1186/s13075-018-1661-5) contains supplementary material, which is available to authorized users.

## Background

Pain affects nearly all patients with chronic inflammatory conditions, including rheumatoid arthritis (RA). Pain in RA is due to both local factors at the level of the joint as well as central neuronal processing. Pain and inflammation interact at many levels from the molecular to the psychological, making the pain of chronic inflammation challenging to manage [1].

Opioids are potent analgesics. Their use for chronic pain is limited, however, by the fact that they provoke pharmacologic tolerance, meaning that higher doses are needed to achieve effective analgesia. These higher doses lead to problems of overdosing and dependence/addiction [2]. Opioids and their receptors are intimately involved in chronic inflammation [3]. For instance, endogenous opioids released from chronic inflammatory cells can provide local pain relief by acting on opioid receptors whose sensitivity is increased by certain inflammatory compounds. Recent studies have demonstrated that certain opioid receptors can form heterodimers with inflammation-promoting chemokine receptors [4].

Numerous chemokines and their receptors have been implicated in leukocyte infiltration into the inflamed synovium of RA patients [5]. The C-C chemokine-receptor type 5 (CCR5) is abundantly expressed on T cells and macrophages, and its ligand (CCL5) is found in the synovial fluid of patients with RA [5]. In juvenile arthritis, elevated synovial levels of CCL5 predict a more severe disease course [6]. These observations suggest that CCR5 could be mechanistically involved in the pathogenesis of inflammatory arthritis. Several studies have evaluated the efficacy of CCR5 antagonists in patients with RA, alone or in combination with methotrexate. However, none of these compounds has resulted in reduced arthritis severity relative to placebo [7–9].

CCR5 and the mu opioid receptor (MOR) are both expressed on certain neurons as well as on certain leukocytes [4]. CCR5 can heterodimerize with MOR, which can lead to functional desensitization of both receptors [10]. Specifically, opioid treatment of immune cells inhibits the chemotactic response induced by several chemokines; conversely, pretreatment with some chemokines reduces the chemotaxis induced by some opioids [10]. Our group has developed a novel bivalent pharmacophore, MCC22, comprising a MOR agonist and a CCR5 antagonist (TAK-220) joined by a 22-atom spacer [11, 12] (Additional file 1: Figure S1). The intention of this design was to target the opioid agonist to anatomic sites of increased CCR5 expression, thereby potentially increasing its therapeutic potency. Indeed, in a model of lipopolysaccharide (LPS)-induced inflammation, MCC22 provided ~ 3500-fold more potent analgesia than did a mixture of its monomer constituents when delivered directly to the central nervous system [11]. In that same model, the irreversible MOR antagonist beta-funaltrexamine (beta-FNA) [13] potently inhibited the antinociceptive efficacy of MCC22, implicating the importance of the MOR agonist pharmacophore of MCC22 in antinociception via the MOR protomer in the MOR-CCR5 heterodimer (Akgün et al., manuscript in preparation). A related bivalent ligand MMG22, comprising a MOR agonist and a metabotropic glutamate receptor 5 (mGluR5) antagonist, also connected by a 22-atom spacer, also has much greater potency than its monomer constituents in a murine cancer model, and importantly did not induce opioid tolerance [14]. The 22-atom spacer length of these bivalent pharmacophores appears to be critical. Longer or shorter spacers have resulted in much lower analgesic efficacy, suggesting receptor heteromers as targets for both MCC22 and MMG22 bivalent ligands [11, 14].

We hypothesized that MCC22 administration would provide potent analgesia in the context of inflammatory arthritis. We tested this hypothesis in the K/B.g7 T-cell receptor (TCR) transgenic mouse model of spontaneous inflammatory arthritis. In this model, mice develop symmetric polyarticular arthritis affecting primarily the ankle/wrist equivalents and the digits [15].

## Methods

### Animals

KRN TCR transgenic mice on the C57BL/6 (B6) background and B6 mice congenic for H-2^g7^ (B6.g7) were gifts from Diane Mathis and Christophe Benoist (Harvard Medical School, Boston, Massachusetts and Institute de Génétique et de Biologie Moléculaire et Cellulaire, Strasbourg, France). *Ccr5*-deficient (B6.129P2-^*Ccr5tm1Kuz/J*^) [16] and wild-type B6 mice were purchased from Jackson Laboratory (Bar Harbor, ME, USA). The mice were intercrossed as needed to create the animals used for this study.

Animals were maintained in our specific pathogen-free animal facility at the University of Minnesota (Minneapolis, MN) under protocols approved by the Institutional Animal Care and Use Committee of the University.

### Mouse genotyping

Genotypes of mice were determined by polymerase chain reaction (PCR) amplification of genomic DNA extracted from mouse ear punched tissues.

### Determination of serum CCL5 levels

Serum concentrations of CCL5/RANTES were measured using a commercially available enzyme-linked immunosorbent assay, according to the manufacturer’s instructions (R&D Systems).

### Immunohistochemical staining

Tissue sections were prepared as previously described [17, 18]. Briefly, paraffin sections of 10% buffered formalin-fixed and decalcified ankles were deparaffinized and placed in acidic antigen retrieval solution (R&D Systems) at 95 °C for 10 min. Sections were cooled at room temperature for 20 min and rinsed under cold tap water for 5 min. Bloxall (Vector Laboratories) was applied to sections to block endogenous peroxidases for 10 min. Fc receptors were blocked with anti-CD16/32 (clone 70–0161-M001, Tonbo Biosciences) and anti-CD64 (clone 139,302, BioLegend) in 2% bovine serum albumin (BSA). Sections were incubated overnight with anti-CCR5-Biotin (clone 107,003, BioLegend) or control Armenian Hamster IgG-Biotin at 4 °C at 1:100 dilution. Sections were incubated with Vectastain ABC reagent (Vector Laboratories) for 30 min followed by ImmPACT DAB peroxidase substrate (Vector Laboratories) for 10 min. Sections were cleared with Xylene and mounted with Vectamount (Vector Laboratories).

### Compound synthesis and administration

MCC22 and MCC14 were synthesized as described previously [11]. Morphine was purchased from Mallinckrodt Pharmaceuticals Company (Mallinckrodt Chemical, St. Louis, Missouri). All the compounds were dissolved in 10% (wt/vol) dimethyl sulfoxide (DMSO) and then diluted to less than 1% DMSO in the final solutions. Vehicle control was 1% DMSO. All the compounds were administrated via intraperitoneal injection.

### Mechanical hypersensitivity test

The mechanical pain threshold was quantified by measuring the hind paw withdrawal response using an electronic Von Frey anesthesiometer (IITC Life Science, Woodland Hills, CA). Briefly, animals were placed into enclosures in a test chamber with a metal mesh floor through which the von Frey rigid tip monofilaments were applied, and mechanical pain thresholds (in grams) were measured. The mechanical threshold (in grams) of both hind paws was averaged for each animal. Baseline measurements were recorded before each dose of drug or vehicle. Animals were tested again for their hind paw withdrawal 2 h after drug administration. Drugs were administered once daily for up to 15 consecutive days. Recordings were taken on days indicated in each Figure. After a maximum of 15 days, drug administration was stopped and the animals were monitored to record the accumulative analgesic effect of the administered drug.

### Tolerance test

A drug tolerance test was performed by administering the ED_90_ dose (defined as the dose at which 90% of animals experience an analgesic effect) of drug twice daily for 9 days. Electronic Von Frey stimulation was performed on days 1, 3, and 9, and the mechanical thresholds were recorded.

### Grip strength measurement

Forelimb grip strength was recorded using a Chatillon Force Gauge DFE II (Ametek) according to the manufacturer’s instructions.

### Nest-building behavior

Scoring of mouse nest building was performed as described previously [19]. Briefly, nest scores (scale of 1–5) are determined by the wall height of nest/dome made by mice around them in the cage using the same amount of nesting material (1 = nesting material scattered in the cage, 2 = flat nest, 3 = less than half height of nesting dome, 4 = half height of nesting dome, 5 = greater than half height of nesting dome). Double-blinded scoring was performed to measure the height of the nesting dome.

### Arthritis assessment

Assessment of ankle thickness and arthritis severity scores were performed as per standard protocols [15]. In brief, ankle thickness measurements were determined by measuring across the malleoli using a Kafer dial thickness gauge with flat anvils (Long Island Indicator Service, Hauppauge, NY). Arthritis severity scores are determined using a scale of 0–3 for each paw, where 0 is normal and 3 is maximum arthritis severity; the maximum total score for a given mouse is 12.

### Histopathology of ankles

Paraffin sections of 10% buffered formalin-fixed and decalcified ankles were stained with hematoxylin and eosin. Sections were scored from 0 for normal to 5 for inflammation, fibroplasia, and cartilage injury per the scoring system of Caplazi and Diehl [20].

### Statistical analysis

Prism 5.01 (GraphPad Software, Inc., La Jolla, CA) was used for all analysis and graphical representation of data. For tests involving multiple comparisons, one-way or repeated measures two-way analysis of variance (ANOVA) were used followed by post-hoc Tukey’s or Sidak’s multiple-comparison tests, as appropriate. For comparisons of two groups, the paired *t* test or Mann-Whitney *U* test was used as appropriate. The particular statistical tests used for each experiment are indicated in the Figure legends. ED_50_ values with 95% confidence intervals (CIs) were computed with Prism using nonlinear regression methods. For all tests, *P* values < 0.05 were considered significant.

## Results

### CCR5/CCL5 expression is increased in K/B.g7 arthritic mice

**Fig. 1:**
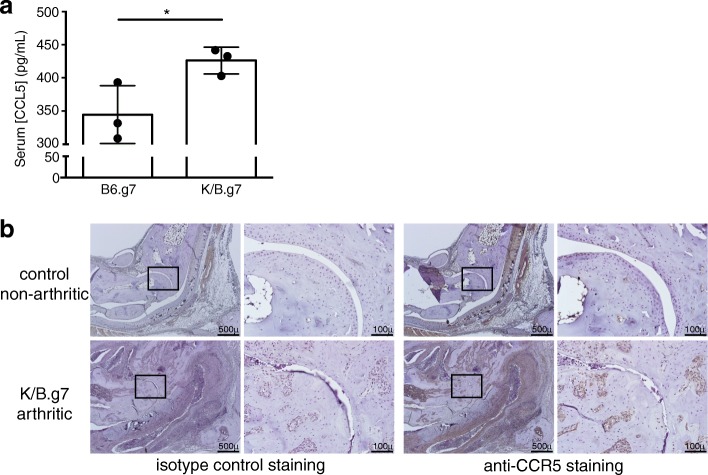
CCL5 and CCR5 expression are increased in K/B.g7 mice. **a** Serum was collected from 8-week-old control nonarthritic B.g7 mice or arthritic K/B.g7 mice (*n* = 3/group). The concentration of CCL5 was determined by ELISA and data were compared using a Mann Whitney *U* test. Points represent individual mice; bars indicate mean ± SEM; *p < 0.05. **b** Sections of ankle joints from nonarthritic control mice or K/B.g7 mice were stained with isotype control antibody or anti-CCR5 antibody, as indicated. Brown indicates positive staining. The black box in the lower-power (4× objective) images indicates the region of the tibiotalar joint magnified (20× objective) in the right images. Relative to the control ankles, ankles from the arthritic mice have joint space narrowing, cartilage loss, and numerous CCR5-expressing inflammatory cells

We first sought to determine whether CCL5 and CCR5 were overexpressed in K/B.g7 mice. Serum levels of the CCR5 ligand CCL5 (RANTES) were higher in K/B.g7 arthritic mice relative to nonarthritic control animals (Fig. 1a). Immunohistochemical staining demonstrated some CCR5 expression near the tendons and blood vessels of nonarthritic control animals; in arthritic animals, infiltrating cells expressing CCR5 are readily identifiable around and within the inflamed ankle joint, including in rests of cells in the subchondral bone (Fig. 1b); cartilage loss and tibiotalar joint space narrowing are also evident.

### MCC22 is 3000-fold more potent than morphine, and depends on the 22-atom spacer length

**Fig. 2:**
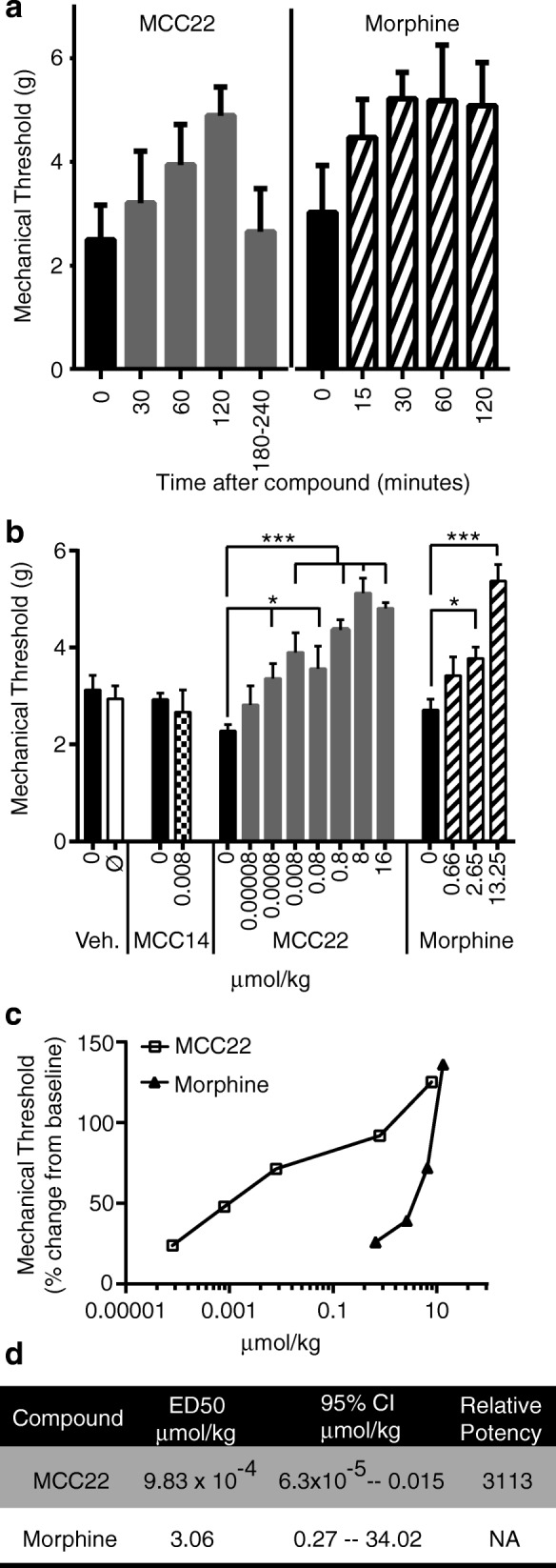
MCC22 is ~ 3000-fold more potent than morphine. **a** Mechanical thresholds were measured at the indicated times after administration of MCC22 (8 μmol/kg) or morphine (13.25 μmol/kg) to arthritic K/B.g7 mice; *n* = 4–10 mice/group, bars represent means ± SD. **b** Arthritic K/B.g7 mice received injections of vehicle, MCC22, MCC14, or morphine intraperitoneally at the indicated doses. The mechanical pain threshold was determined 2 hours later for vehicle, MCC22, MCC14, and 30 min later for morphine. Each bar includes data from 5 to 12 mice. The data were compared within each group using either paired *t* tests (for vehicle and MCC14) or one-way ANOVA followed by post-hoc Tukey’s test (for MCC22 and morphine). Data are presented as mean ± SEM; *p < 0.05, ***p < 0.001, versus the baseline for the group. **c** The mean data are displayed as a percent change from baseline. **d** The calculated ED_50_ of each compound is shown, along with the 95% confidence interval (CI)

We next asked whether the bivalent pharmacophore, MCC22, comprising a MOR agonist and CCR5 antagonist, would provide effective analgesia in this model. We evaluated the mechanical pain threshold in mice using an electronic Von Frey test. We first determined the time to peak analgesic effect for both MCC22 and the commonly used opioid receptor agonist morphine. MCC22 reached peak analgesic effect at 2 h and morphine at 30 min after administration (Fig. 2a); we therefore used these peak times for all subsequent experiments. We next compared the potency of MCC22 to morphine. A standard dose response experiment revealed that MCC22 was ~ 3000-fold more potent than morphine in alleviating mechanical hyperalgesia in arthritic K/B.g7 mice (Fig. 2b–d). The 22-atom spacer length of MCC22 was crucial—a similar compound with a shorter 14-atom spacer (MCC14) did not provide analgesia when given at a similar dose (Fig. 2b).

### MCC22 produces cumulative analgesia in the setting of chronic inflammation

**Fig. 3:**
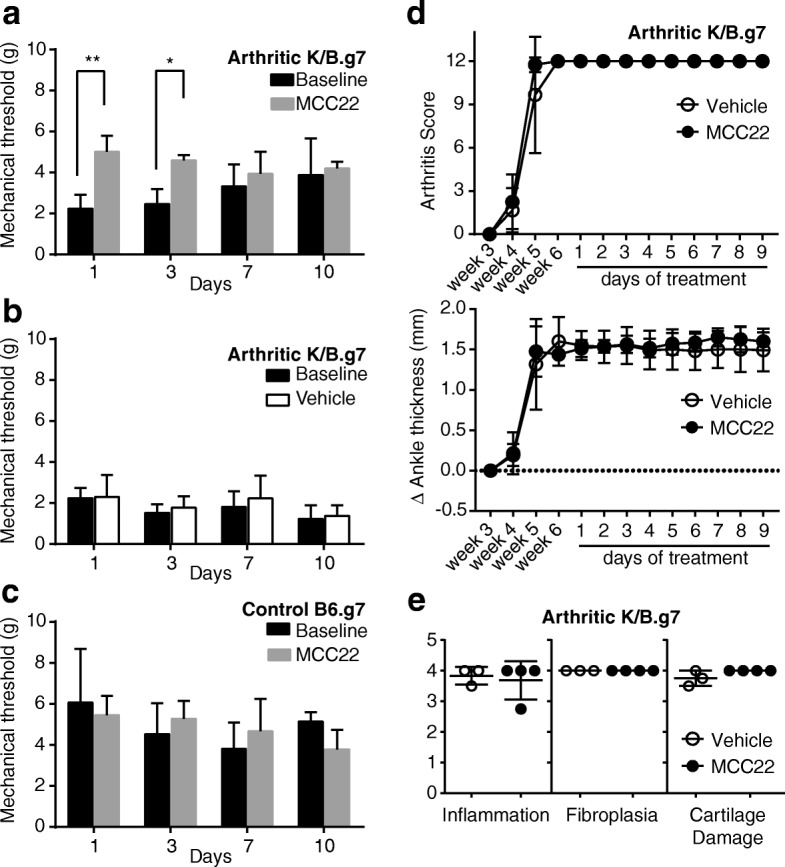
MCC22 is effective in the setting of inflammation, has an accumulative effect, and depends on the 22-atom spacer length. **a** Six- to 8-week-old K/B.g7 arthritic mice were treated with MCC22 once daily (8 μmol/kg/dose). The baseline mechanical pain threshold was recorded each day. The drug was then administered, and the pain threshold was determined again 2 h later. **b** K/B.g7 arthritic mice that received saline injections showed no increase in mechanical pain threshold. **c**. Nonarthritic control B6.g7 mice have higher baseline mechanical pain thresholds than K/B.g7 mice (compare with panels **a** and **b**) and this is not increased by MCC22 (dosed as in panel **a**). Data were analyzed using two-way repeated-measures ANOVA with post-hoc Sidak’s multiple comparisons test. Data are displayed as mean ± SEM; *n* = 4–5 mice/group. **d** Arthritic K/B.g7 mice received MCC22 or vehicle once daily starting at 6 weeks of age. MCC22 did not affect arthritis severity or ankle thickness. Data are representative of three experiments with a total of 11–13 mice/group and are displayed as mean ± SD. **e** Histopathologic scoring of ankle inflammation, fibroplasia, and cartilage damage following treatment with vehicle or MCC22. Circles indicate individual mice, bars indicate mean ± SD. *p < 0.05, **p < 0.01

To determine the efficacy of MCC22 in chronic arthritis pain, we used K/B.g7 mice that were 6 weeks old and had maximum arthritis severity scores of 12. Daily administration of MCC22 to K/B.g7 arthritic mice resulted in higher analgesic potency at days 1 and 3 relative to the daily baseline pain threshold (Fig. 3a). It is noteworthy that the baseline pain threshold in MCC22-treated arthritic mice increased steadily from days 1 to 10, with 110%, 148%, and 173% increases over the day 1 thresholds on days 3, 7, and 10, respectively (Fig. 3a). Arthritic mice that received vehicle had no change in the pain threshold (Fig. 3b). Control nonarthritic mice had higher baseline pain thresholds than arthritic mice, and this was not further increased by MCC22, even with a dose of MCC22 that is 10^4^-fold greater than its ED_50_ (Fig. 3c). These findings demonstrate that MCC22 provides effective analgesia in the setting of chronic inflammatory arthritis. Even at this high dose, MCC22 also did not affect arthritis severity scores; all mice continued to have maximum arthritis scores of 12 throughout the duration of the experiment (Fig. 3d). Furthermore, MCC22 treatment did not affect the degree of ankle thickening (Fig. 3d) or histopathologic scoring of arthritis severity (Fig. 3e and Additional file 1: Figure S2).

### MCC22 does not induce opioid tolerance

**Fig. 4:**
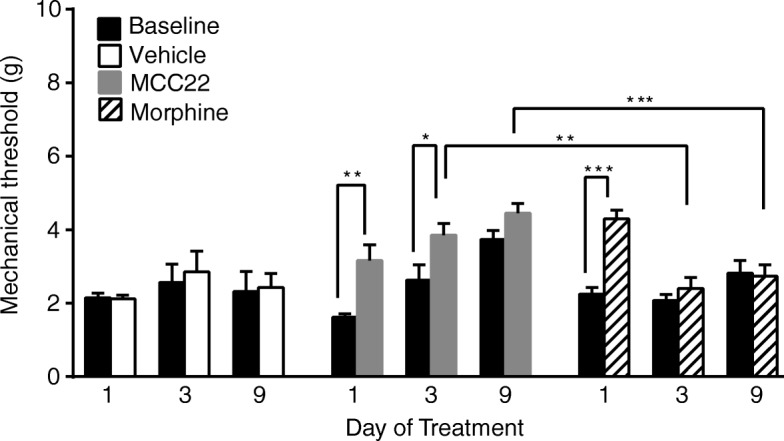
MCC22 does not induce pharmacologic tolerance. Arthritic K/B.g7 mice were treated daily with vehicle, MCC22 (0.008 μmol/kg/dose), or morphine (6.62 μmol/kg/dose). Mechanical pain thresholds were determined daily before (baseline) and after the administration of the indicated compound. The analgesic effect of MCC22 is apparent at days 1 and 3; in addition, the baseline pain tolerance increases in this group. In contrast, the analgesic efficacy of morphine is lost by day 3. Data are displayed as mean ± SEM; *n* = 4–10 mice/group. Data were analyzed using repeated measures two-way ANOVA with post-hoc Tukey’s test for multiple comparisons within treatment groups and Sidak’s test for multiple comparisons between treatment groups. *p < 0.05, **p < 0.01, ***p < 0.001

A major limitation to the clinical use of morphine and other opioids is that they induce pharmacologic tolerance. We therefore evaluated whether MCC22 induced tolerance in this model, using morphine as a comparator. MCC22 given at 70% of the maximum pharmacologic effect (MPE) dose retained analgesic efficacy over 9 days of administration, whereas the efficacy of morphine was diminished as early as day 3 of administration (Fig. 4). Furthermore, prolonged administration of morphine did not increase the baseline pain threshold, whereas MCC22 did (Figs. 2a and 4). Thus, MCC22 is substantially more potent than morphine and also does not cause typical opioid tolerance.

### MCC22 improves function in arthritic mice

**Fig. 5:**
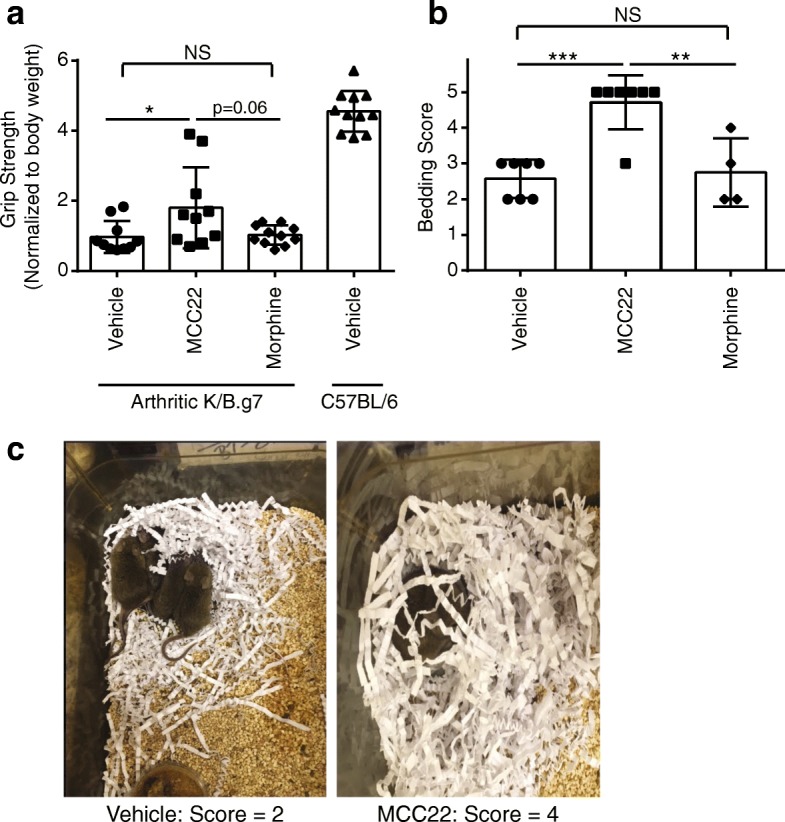
MCC22 improves grip strength and nest-building behavior. **a** Grip strength was measured in arthritic K/B.g7 mice or nonarthritic control mice. The K/B.g7 mice were treated with the indicated compounds (MCC22 0.008 μmol/kg/dose and morphine 6.62 μmol/kg/dose). Data were compared using one-way ANOVA with post-hoc Tukey’s test for multiple comparisons and using the C57BL/6 group as the reference group. The values for the arthritic K/B.g7 mice were all significantly lower than that of the nonarthritic C57BL/6 mice (*p* < 0.0001). **b** Nest-bedding scores were recorded among groups of arthritic K/B.g7 mice treated with the indicated compounds. Data were compared using one-way ANOVA with post-hoc Tukey’s test for multiple comparisons. In **a** each point represents one mouse, and in **b** each point represents one cage of mice. Bars represent mean ± SEM. *p < 0.05, **p < 0.01, ***p < 0.00,1 NS not significant. **c** Representative photographs of arthritic K/B.g7 mice demonstrating a bedding score of 2 for mice treated with vehicle (left) or and a bedding score of 4 for mice treated with MCC22 (right)

Patients with inflammatory arthritis experience difficulties with routine activities of daily living, due to both pain and restricted mobility of joints. We therefore asked whether MCC22 improved functional measures in K/B.g7 arthritic mice. First, we evaluated forelimb grip strength. Compared to control nonarthritic mice, all of the K/B.g7 mice had lower grip strength. Grip strength improved in K/B.g7 mice treated for 9 days with MCC22, but not in mice treated with morphine (Fig. 5a). This difference was not apparent at earlier time points in the experiment (data not shown). As a second measure of function, we evaluated nesting behavior. Healthy mice normally build nests when given pressed nesting material, and nesting behavior can be quantified using a standard scoring scale. Indeed, we found that control nonarthritic mice built more robust nests than did arthritic K/B.g7 mice. MCC22 treatment of K/B.g7 mice normalized the nesting behavior, whereas morphine had no effect (Fig. 5b, c). In summary, MCC22 improves functional measures in mice with inflammatory arthritis. These effects were not due to reduced inflammation, as treatment with MCC22 had no effect on arthritis severity (see Fig. 3d, e). Furthermore, these improved functional measures are consistent with our observations that MCC22 did not induce typical opioid side effects (e.g., apnea, sedation), even at higher doses.

### Efficacy of MCC22 depends on CCR5

**Fig. 6:**
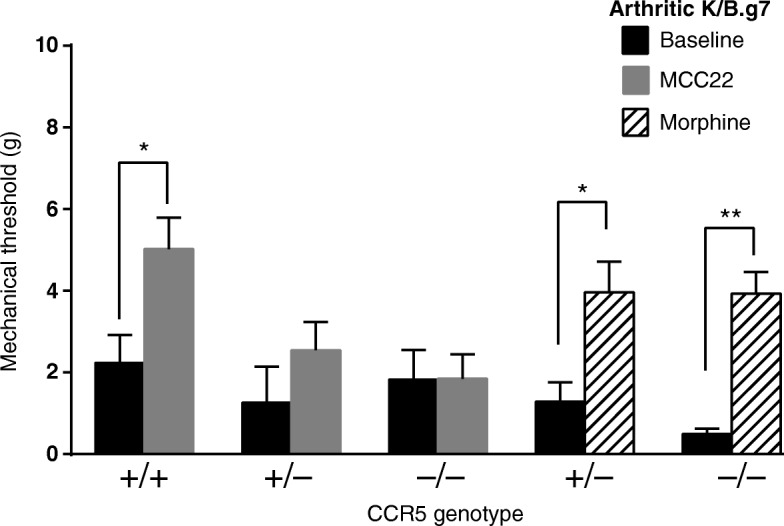
The activity of MCC22 depends on CCR5 expression. MCC22 (0.008 μmol/kg/dose) or morphine (6.62 μmol/kg/dose) were administered to CCR5^+/+^, CCR5^+/−^ or CCR5^−/−^ K/B.g7 mice, and the mechanical pain threshold was determined before and after administration of the compound. MCC22 is not effective in CCR5-deficient K/B.g7 mice, whereas morphine continues to provide effective analgesia. Data were compared within each group using paired *t* tests and are displayed as mean ± SEM; *n* = 4–7 mice/group. *p < 0.05, **p < 0.01

MCC22 comprises a CCR5 antagonist and MOR agonist pharmacophores. To test its binding specificity, we evaluated the analgesic efficacy of MCC22 in K/B.g7 mice genetically lacking CCR5. Of note, CCR5-deficient K/B.g7 mice developed arthritis equivalently to wild-type K/B.g7 mice (data not shown). In CCR5-deficient K/B.g7 mice, MCC22 provided no analgesic effect, whereas the analgesic efficacy of morphine remained intact (Fig. 6). These data demonstrate that the potent analgesic effect of MCC22 requires CCR5.

## Discussion

Opioids are potent analgesics for inflammatory arthritis and other conditions. However, chronic opioid administration promotes downregulation of opioid receptors and hyperalgesia that leads to reduced efficacy. Furthermore, because of their addictive and dependent properties, misuse of prescription opioids has massively increased in recent years. Thus, there is an intense demand to develop safer alternatives to manage pain in patients with arthritis.

Chemokines are known to mediate and modulate pain pathways [21, 22]. Blockade of chemokine-mediated signaling pathways is emerging as a new potential treatment for alleviating chronic pain. A strong correlation has been reported between numerous chemokine pathways and inflammatory arthritis severity, including CCR5 [4]. Consistent with this, we observed increased CCR5 expression in the synovial joints of K/B.g7 arthritic mice, along with an elevated circulating concentration of its ligand CCL5/RANTES. We therefore reasoned that the CCR5 antagonist moiety within MCC22 might direct it specifically to cells mediating inflammatory pain.

We have demonstrated that MCC22 is ~ 3000-fold more potent than morphine, works specifically in the setting of inflammation, and does not induce typical opioid tolerance. We also observed a steady increase in baseline pain thresholds with daily MCC22 administration, but not with morphine. Furthermore, we demonstrated that MCC22 significantly improved the routine functional activities of arthritic mice, with improved grip strength and improved nest-building behavior. Taken together, these findings suggest that MCC22 could be a useful analgesic for patients with chronic inflammatory pain, without inducing tolerance/dependence.

Importantly, MCC22 did not reduce the severity of arthritis in this model. This is consistent with our finding that CCR5-deficient K/B.g7 mice still developed arthritis equivalent to control K/B.g7 mice and prior reports that CCR5 deficiency does not affect the severity of serum-transferred arthritis in mice [23]. It is also consistent with studies of CCR5 antagonists (maraviroc, AZD5672, and SCH351125) in patients with RA, which have failed to demonstrate improvement in disease activity scores [7–9]. Thus, the main benefit of the CCR5 antagonist moiety of MCC22 in this system is to improve the analgesic potency of its MOR agonist moiety, likely by engaging MOR-CCR5 heterodimers. Consistent with this notion, we have demonstrated that the analgesic potency of MCC22 depends specifically on the 22-atom spacer separating the CCR5 antagonist from the MOR agonist, suggesting that MCC22 interacts with MOR and CCR5 heterodimers in a specific orientation [11]. Additional studies of how the interaction of MCC22 with both MOR and CCR5 modulates the function of both receptors will be critical to understanding the basis for its potency.

The question of whether the main cellular targets of MCC22 are present in nociceptive neurons, microglia, or potentially leukocytes remains open. We favor the possibility that MCC22 is acting on neurons on which upregulation of CCR5 has occurred due to chronic inflammation. Further studies will be needed to test this hypothesis. If neurons are the primary cellular target of MCC22, determining whether it acts on peripheral or central neurons will also be of interest.

In addition to MCC22, several other bivalent analgesic compounds have been described recently, including MDAN-21 (MOR agonist:delta opioid receptor antagonist) [24], a series of MOR agonist:cannabinoid receptor 1 (CB1) antagonists [25], and MMG22 (MOR:metabotropic glutamate receptor 5 (mGluR5) antagonist) [14]. Each of these bivalent compounds has demonstrated antinociceptive activity in different murine models of chronic pain. Similar to MCC22, these compounds also provoke less or no tolerance or addictive behaviors when compared with classical opioids.

In a recent study of mice with arthritis induced by injection of complete Freund’s adjuvant, a novel modified opioid drug, NFEPP ((±)-*N* (3-fluoro-1-phenethylpiperidin-4-yl)-*N*-phenylpropionamide), a fluorinated fentanyl analogue, was reported to have potent analgesic properties [26]. We have observed similar analgesic potency with MCC22 at ~ 1000-fold fold lower dose. Similar to NFEPP, MCC22 also did not exert activity in noninflamed animals. Furthermore, NFEPP did not produce any adverse side effects such as reward-seeking behavior, motor impairment, sedation, respiratory depression, or constipation. MCC22 similarly produced no condition-place preference, a measure of reward-seeking, in a different murine model of pain (our unpublished data). Thus, in the setting of arthritis, MCC22 appears to have a similar analgesic and low side-effect profile to NFEPP, but with greater potency.

## Conclusions

In conclusion, MCC22 is a bivalent CCR5 antagonist and MOR agonist compound that provides potent analgesia in mice with inflammatory arthritis without the typical detrimental side effects of opioids. Ongoing studies are directed at understanding its cellular site(s) of action and how its interaction with CCR5 and MOR generates such remarkable potency.

## Additional file

Additional file 1: **Figure S1.** Structure of MCC22. MCC22 is a bivalent pharmacophore comprising a mu opioid receptor (MOR) agonist and a CCR5 antagonist joined by a 22-atom spacer. **Figure S2.** Representative histologic sections. The images show histologic sections of ankles from nonarthritic control animals and arthritic K/B.g7 mice treated with vehicle (DMSO) or daily MCC22 for 9 days (8 μmol/kg/dose). Photomicrographs were obtained with a 4× objective. Scale bars = 500 μm. (PDF 3747 kb)

## Abbreviations

MOR: Mu opioid receptor
NFEPP: (±)-*N* (3-fluoro-1-phenethylpiperidin-4-yl)-*N*-phenylpropionamide
RA: Rheumatoid arthritis
TCR: T-cell receptor
