# Supplementary material for: A bivalent compound targeting CCR5 and the mu opioid receptor treats inflammatory arthritis pain in mice without inducing pharmacologic tolerance
Source: Arthritis Res Ther. 2018 Jul 27;20:154. doi: 10.1186/s13075-018-1661-5 (PMC6062996; doi:10.1186/s13075-018-1661-5)

Supplemental Figures

Dutta, R. et al.

A bivalent compound targeting CCR5 and the mu opioid receptor treats inflammatory arthritis pain in mice without inducing pharmacologic tolerance.

Supplemental Figure Legends

Supplemental Figure 1: Structure of MCC22. MCC22 is a bivalent pharmacophore comprising a mu opioid receptor (MOR) agonist and a CCR5 antagonist joined by a 22-atom spacer.

Supplemental Figure 2: Representative histologic sections. The images show histologic sections of ankles from non-arthritic control animals and arthritic K/B.g7 mice treated with vehicle (DMSO) or daily MCC22 for 9 days (8 μmol/kg/dose). Photomicrographs were obtained with a 4x objective. Scale bars indicate 500 microns.

Supplemental Figure 1

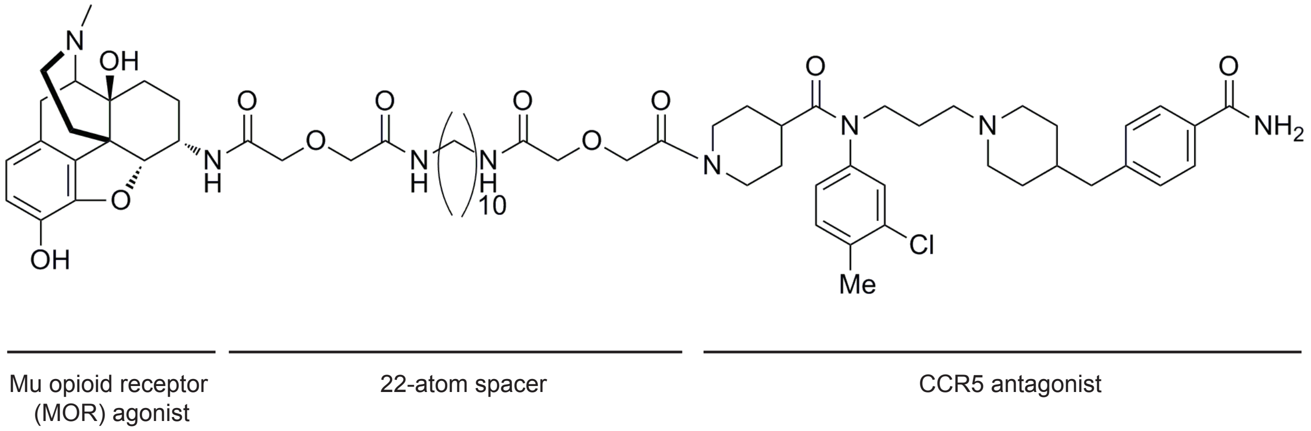

Supplemental Figure 2

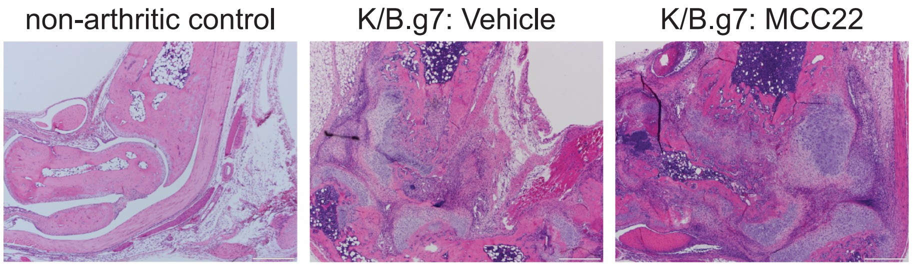

Supplement: Supplementary file 1 — Figure S1. Structure of MCC22. MCC22 is a bivalent pharmacophore comprising a mu opioid receptor (MOR) agonist and a CCR5 antagonist joined by a 22-atom spacer. Figure S2. Representative histologic sections. The images show histologic sections of ankles from nonarthritic control animals and arthritic K/B.g7 mice treated with vehicle (DMSO) or daily MCC22 for 9 days (8 μmol/kg/dose). Photomicrographs were obtained with a 4× objective. Scale bars = 500 μm. (PDF 3747 kb) [file 13075_2018_1661_MOESM1_ESM.pdf]
